# Supplementary material for: Loss of embryonic neural crest derived cardiomyocytes causes adult onset hypertrophic cardiomyopathy in zebrafish
Source: Nat Commun. 2018 Nov 2;9:4603. doi: 10.1038/s41467-018-07054-8 (PMC6214924; doi:10.1038/s41467-018-07054-8)
Supplement: Supplementary file 1 — Supplementary Information [file 41467_2018_7054_MOESM1_ESM.pdf]

Supplementary Information

Loss of embryonic Neural Crest derived Cardiomyocytes causes adult onset hypertrophic cardiomyopathy in zebrafish

Abdul-Wajid et al.

Supplementary Figure 1.

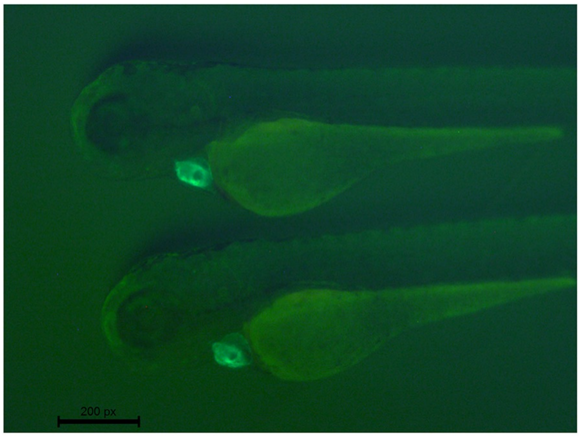

Supplementary Figure 1. *Cm:KillSwitch* transgenic embryos. Whole mount fluorescent images of 4dpf *Cm:KillSwitch* transgenic embryos showing exclusive GFP fluorescence in the heart. Scale bar = 200px.

Supplementary Figure 2.

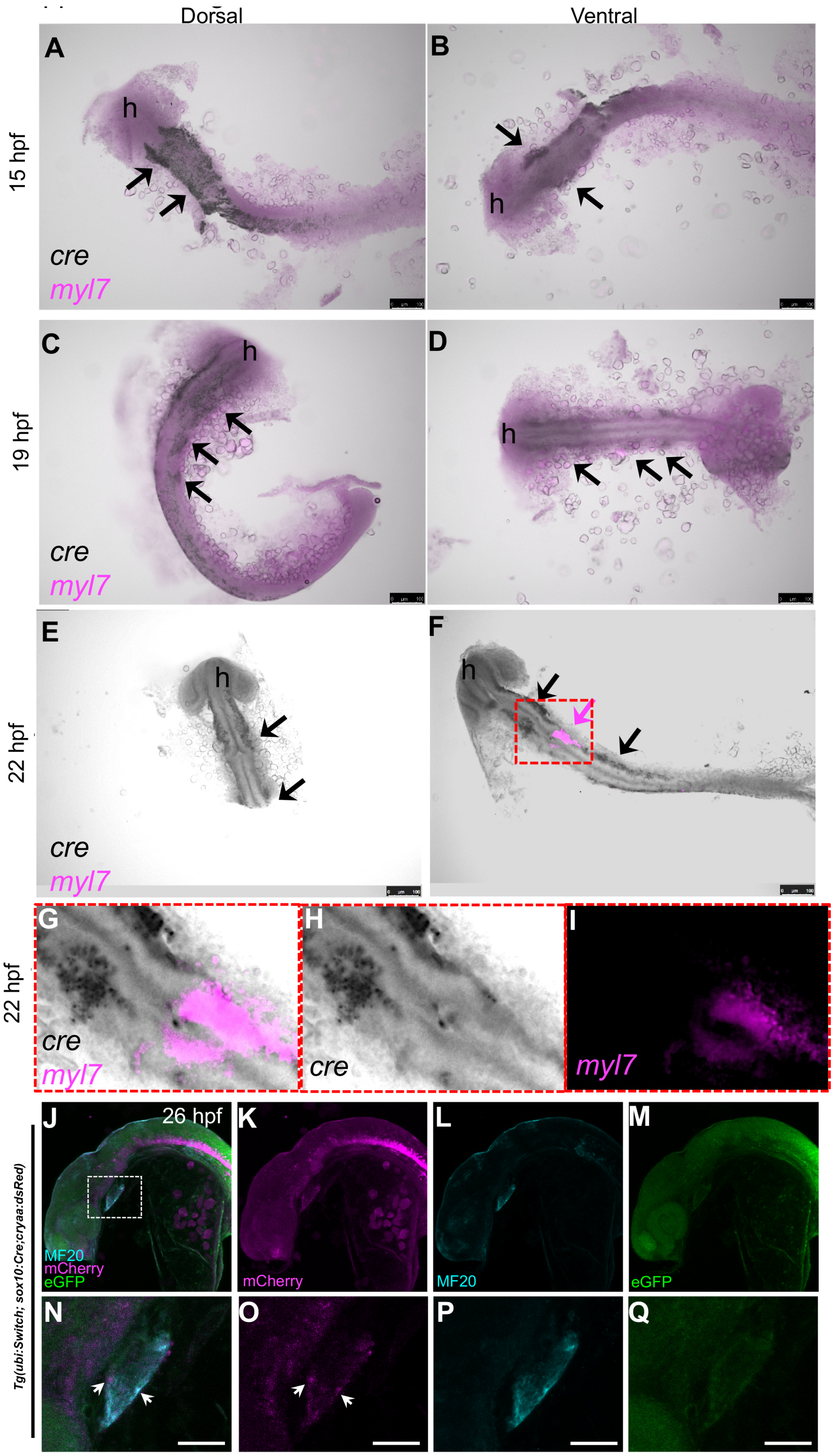

Supplementary Figure 2. *Tg*(*sox10*:*Cre;cryaa:dsRed*) expression relative to the developing heart. A-I) 15hpf, 19hpf and 22hpf embryos from the *Tg*(*sox10*:*Cre;cryaa:dsRed*) line were probed for *myl7* and *cre* RNA detection by two color in-situ hybridization. *myl7* was detected by FastRed staining (fluorescence in Red channel, magenta) and *cre* by NBT-BCIP stain (black). Arrows point to *cre* expression in the stereotypical neural crest lineage pattern progressing medially from the dorsal neural tube. Magenta arrow demonstrates the first detection of the *myl7*+ heart field in the ventral side of 22hpf embryos (low level magenta in A-D was equivalent to background and negative control stained embryos). A few black *cre*+ cells can be seen nearby the *myl7* staining at this stage (black arrows). 'h'= head region of embryo. Yolks were removed to mediate visualization. Scale bar = 100uM. Right panels are the ventral side images of the same embryos in left panels. G-I) Higher magnification of heart field, from red box shown in F. H and I show separate channels for *cre* and *myl7*, respectively. J-Q) 26hpf embryos from a cross of *Tg*(*sox10*:*Cre;cryaa:dsRed*) to the *Tg*(*ubi:Switch*) line were stained for MF20 and imaged for GFP, mCherry and MF20. The bottom panels are close up images of the heart area indicated by white dashed box in J. mCherry signal indicates the Cre switched neural crest cells. A few mCherry positive cells overlap with the MF20 heart marker indicating the first appearances of NC-Cms (white arrows). Scale bar = 75uM in prime labelled panels.

Supplementary Figure 3.

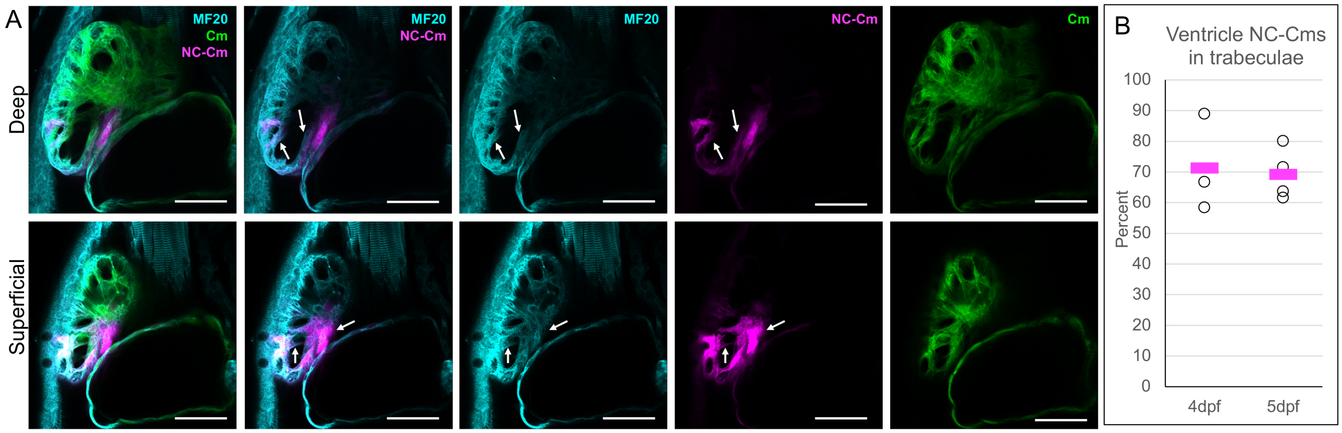

Supplementary Figure 3. A) Cardiomyocyte labelling of *Tg*(Cm:KillSwitch) crossed to *Tg*(*sox10*:*Cre;cryaa:dsRed*). 5dpf embryos were immunostained with anti-sarcomere myosin heavy chain antibody MF20 and imaged in conjunction with tagRFP and GFP fluorescence. Confocal slices of deep (top panel) and superficial (bottom panel) areas of the heart are shown. Arrows point to examples of MF20 stain overlap with the NC-Cm tagRFP fluorescence. Scale bar = 50uM. B) Quantification of the percent of NC-Cms found in trabeculae of the ventricle relative to total ventricle NC-Cms. tagRFP+ NC-Cms were counted from 4 and 5dpf ventricles and designated as being within a trabeculae or not. The number found within trabeculae was divided by the total NC-Cms in the ventricle to yield percentage contribution. Circles represent individual hearts that were counted and bars represent mean of individual data points.

Supplementary Figure 4.

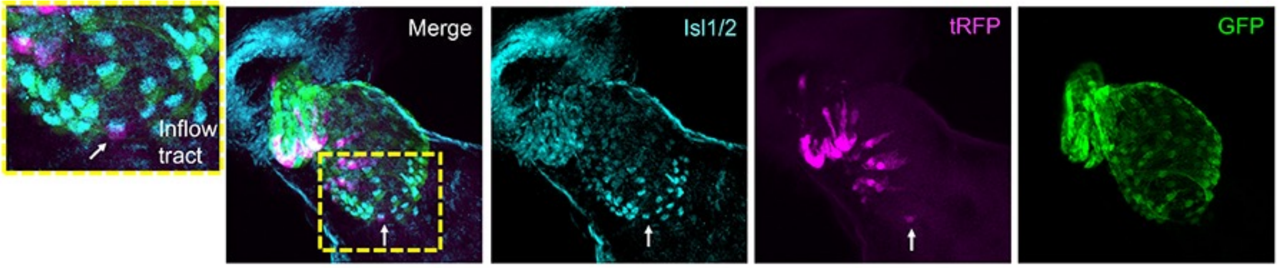

Supplementary Figure 4. Isl1/2 antibody of anterior second heart field and lineage-labeling of NC-Cms indicates interdigitation of neural crest cells into anterior second heart field. Arrow indicates single NC-Cm in the inflow tract that is positive for Isl1/2, also a marker of pacemaker cells[1]. tagRFP and GFP were detected by immunolabeling.

Supplementary Figure 5.

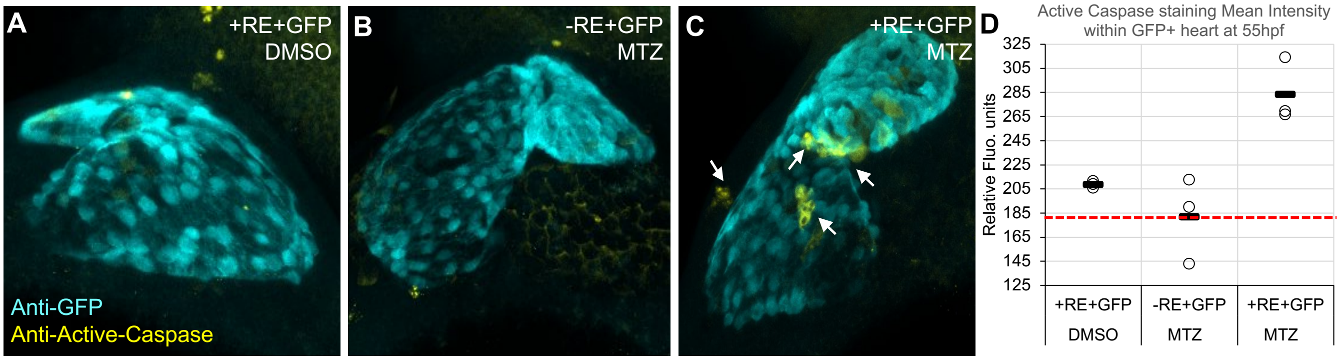

Supplementary Figure 5. Cell death in NC-Cms after MTZ treatment. Embryos derived from transgenic crosses as described in Figure 1 and 2A. A) Control: DMSO treated double transgenic siblings. B) Control: MTZ treated single transgenic siblings. C) MTZ treated double transgenic (+RE+GFP) had positive staining for Active Caspase 3, indicative of cell death (arrows), that was not observed in hearts from controls. D) Quantitation of active Caspase staining signal in control and ablated embryos at 55hpf. Bars indicate mean of n=3 individual heart images (open circles). Red dashed line indicates background level of fluorescent signal in detection channel for active Caspase (179 RFU).

Supplementary Figure 6.

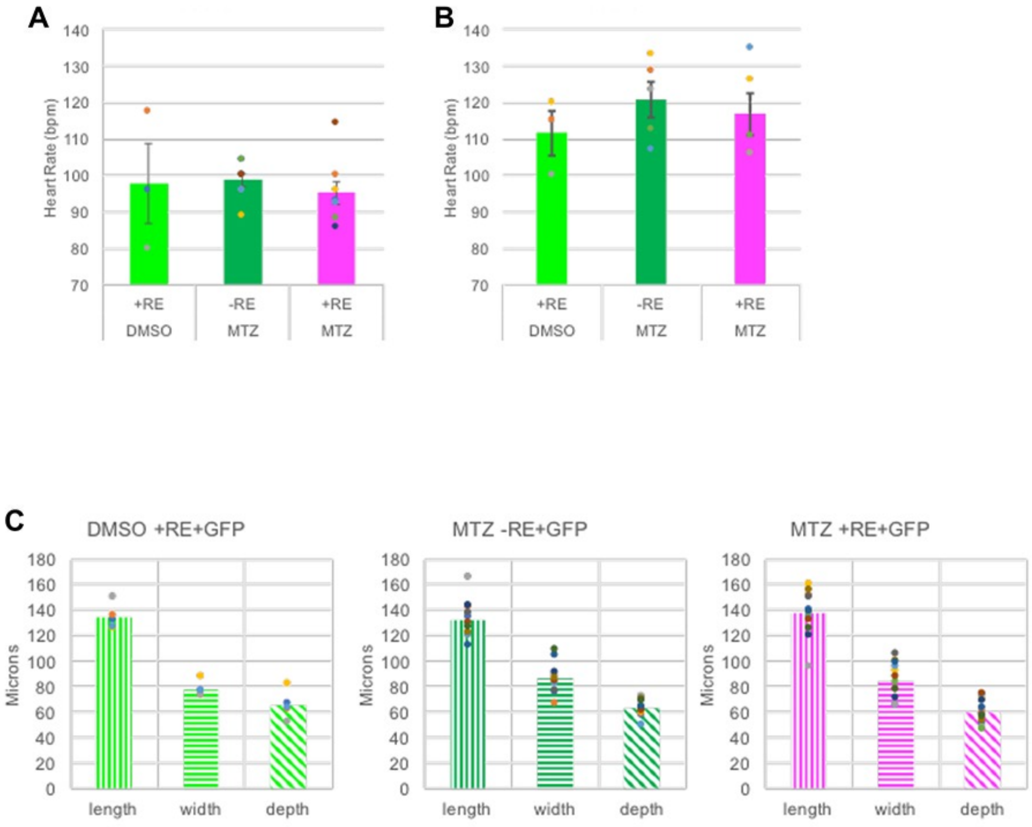

Supplementary Figure 6. NC-Cm ablation has no significant effect on embryonic and juvenile heart rate or size. Heart rate (A) in 6dpf embryos and (B) in 14dpf juveniles. C) Ventricle dimension measurements from controls and MTZ treated siblings at 5dpf.

Supplementary Figure 7.

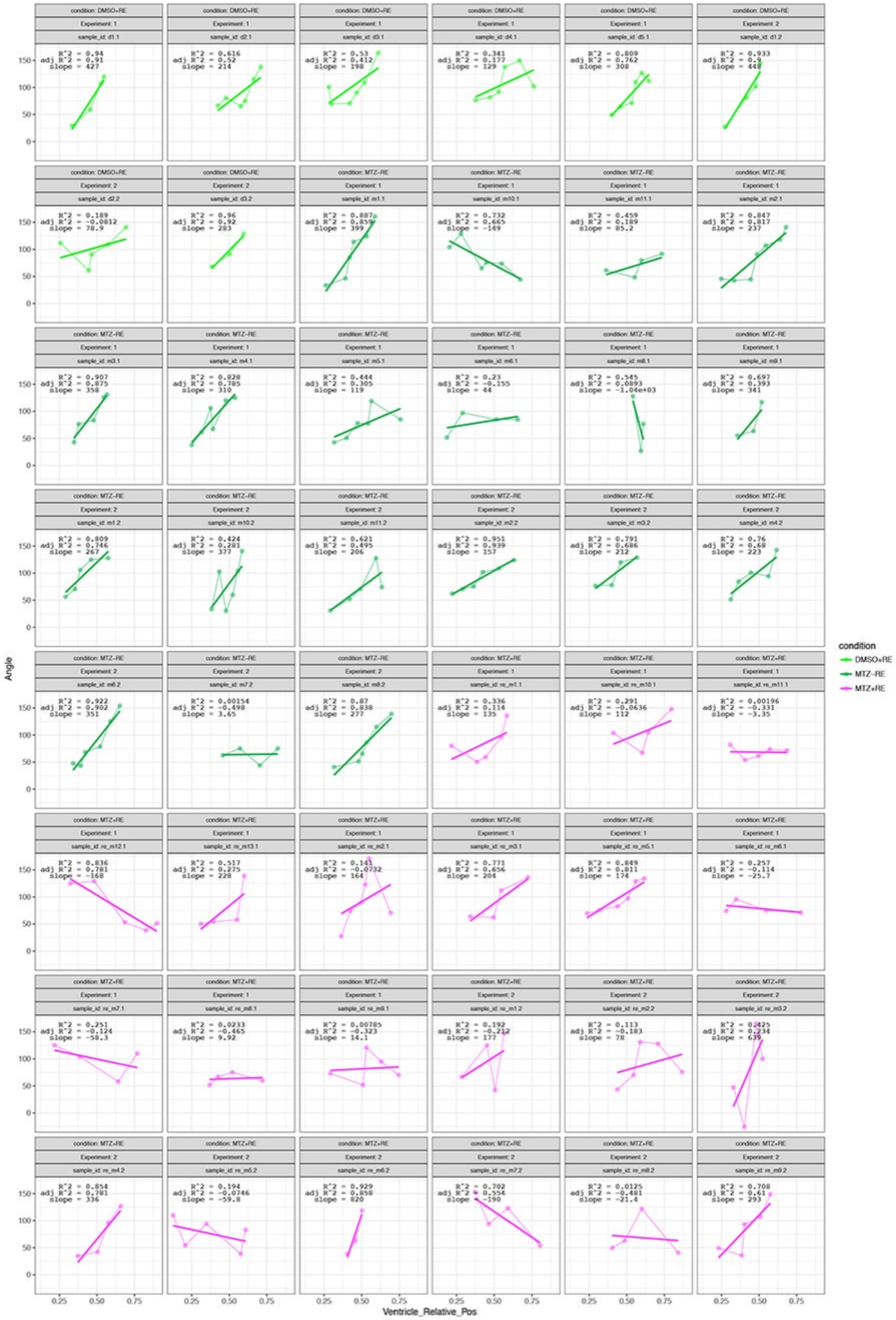

Supplementary Figure 7. Slope calculations from angle and anterior-posterior position measurements of trabeculae in individual hearts from experiments described in Figure 2. Each plot is from an individual heart. Light green is DMSO +RE+GFP, dark green is MTZ –RE+GFP and magenta is MTZ +RE+GFP conditions. Data shown are from one biological replicate. Similar quantification was done for two other biological replicates and shown in sum in Figure 2F.

Supplementary Figure 8.

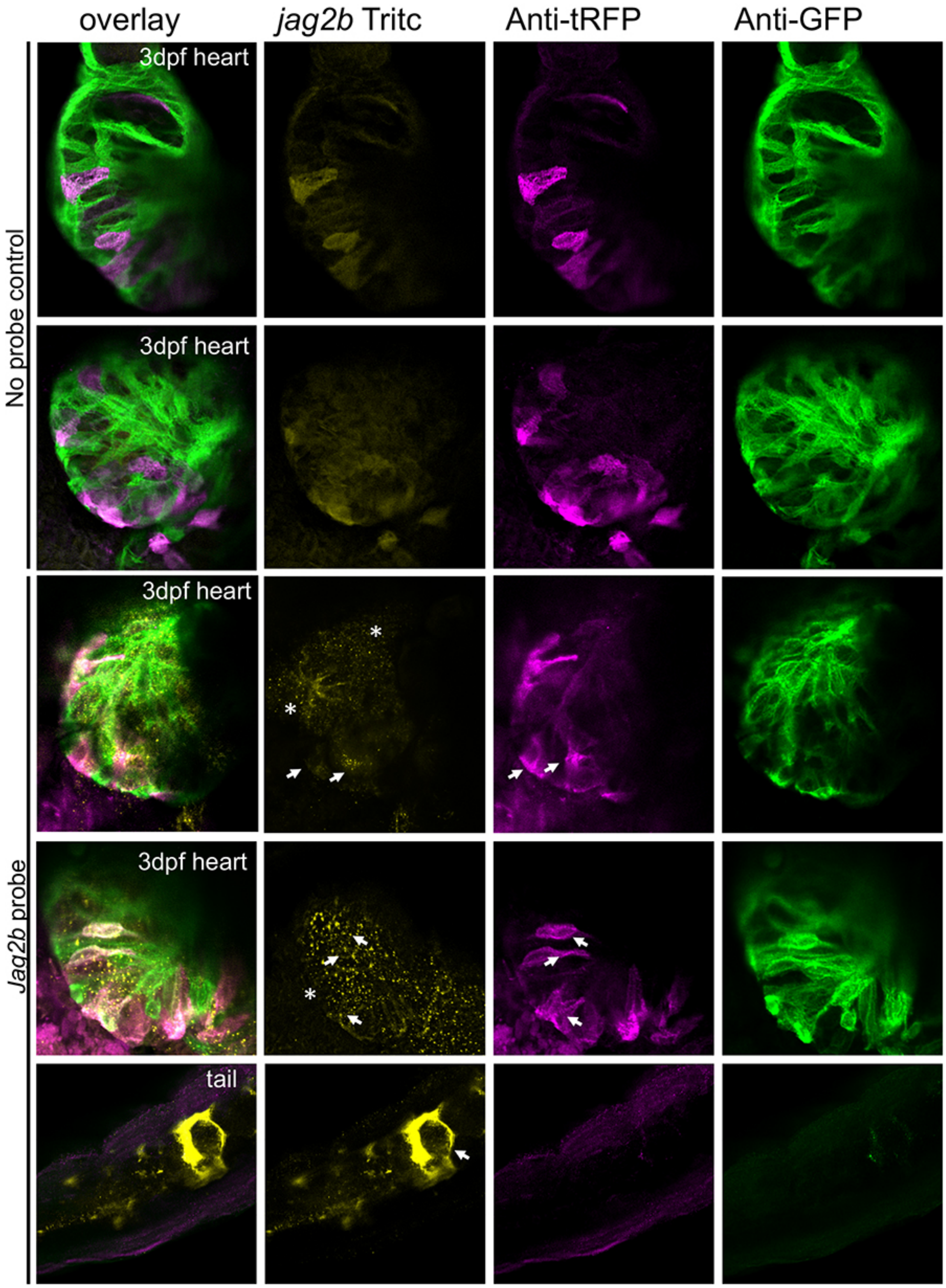

Supplementary Figure 8. *Jag2b* fluorescent in-situ hybridization in NC-Cm labelled embryos. 3dpf hearts from NC-Cm labelled embryos were subject to the fluorescent in-situ protocol with and without *jag2b*-TRITC probe as well as immunostaining for tRFP and GFP. Remnant RFP expression from the NC-Cms is present in the tritc channel however distinct from *jag2b* probe detection (white arrows and astericks compared to no-probe controls). White arrows show NC-Cm specific colocalization of *jag2b* signal and asterisks show other Cm expression or non-Cm expression. The strong tail expression of *jag2b* was used as a positive control for the fluorescent in-situ hybridization experiment.

Supplementary Figure 9.

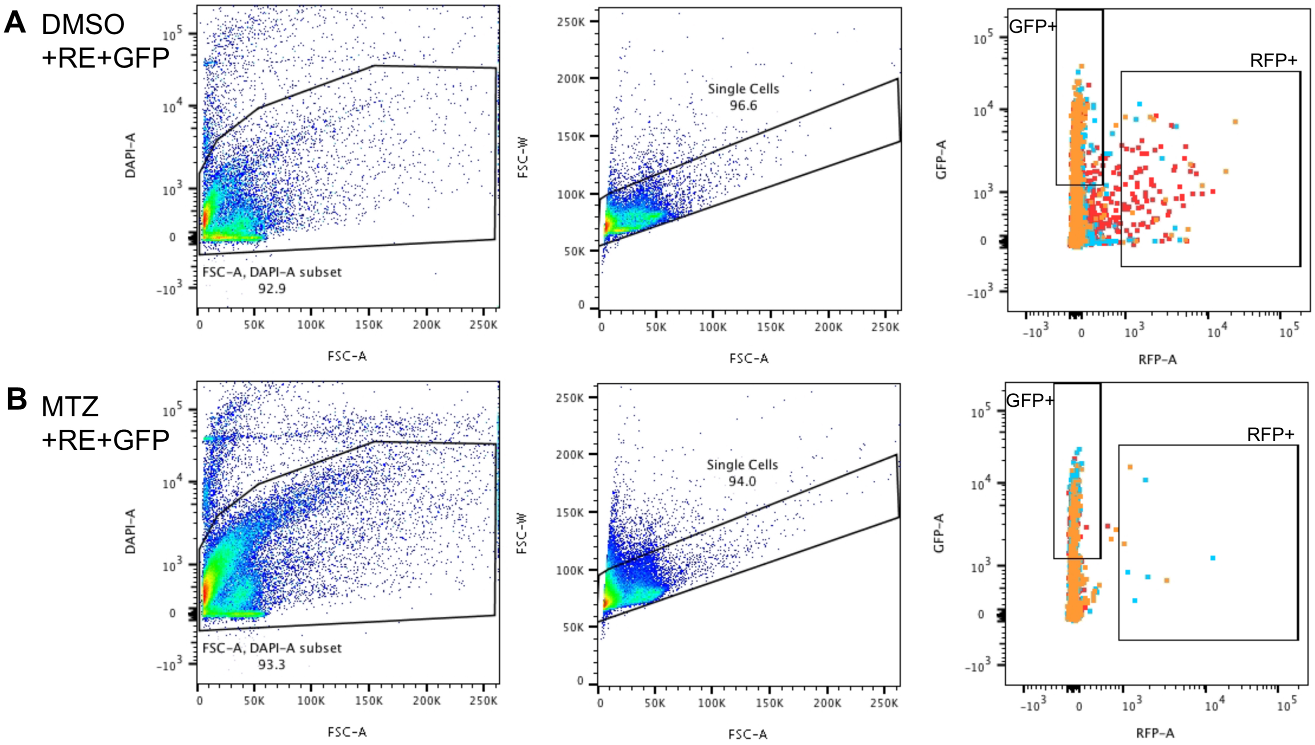

Supplementary Figure 9. FACS analysis of adult hearts post embryonic NC-Cm ablation. A) Control (DMSO +RE+GFP) hearts analyzed for GFP and RFP positive cells. Different colors (orange, blue, red) represent individual hearts analyzed. B) NC-Cm ablated hearts (MTZ +RE+GFP) analyzed by FACS for GFP and RFP positive cells. Different colors (orange, blue, red) represent individual hearts analyzed.

Supplementary Figure 10.

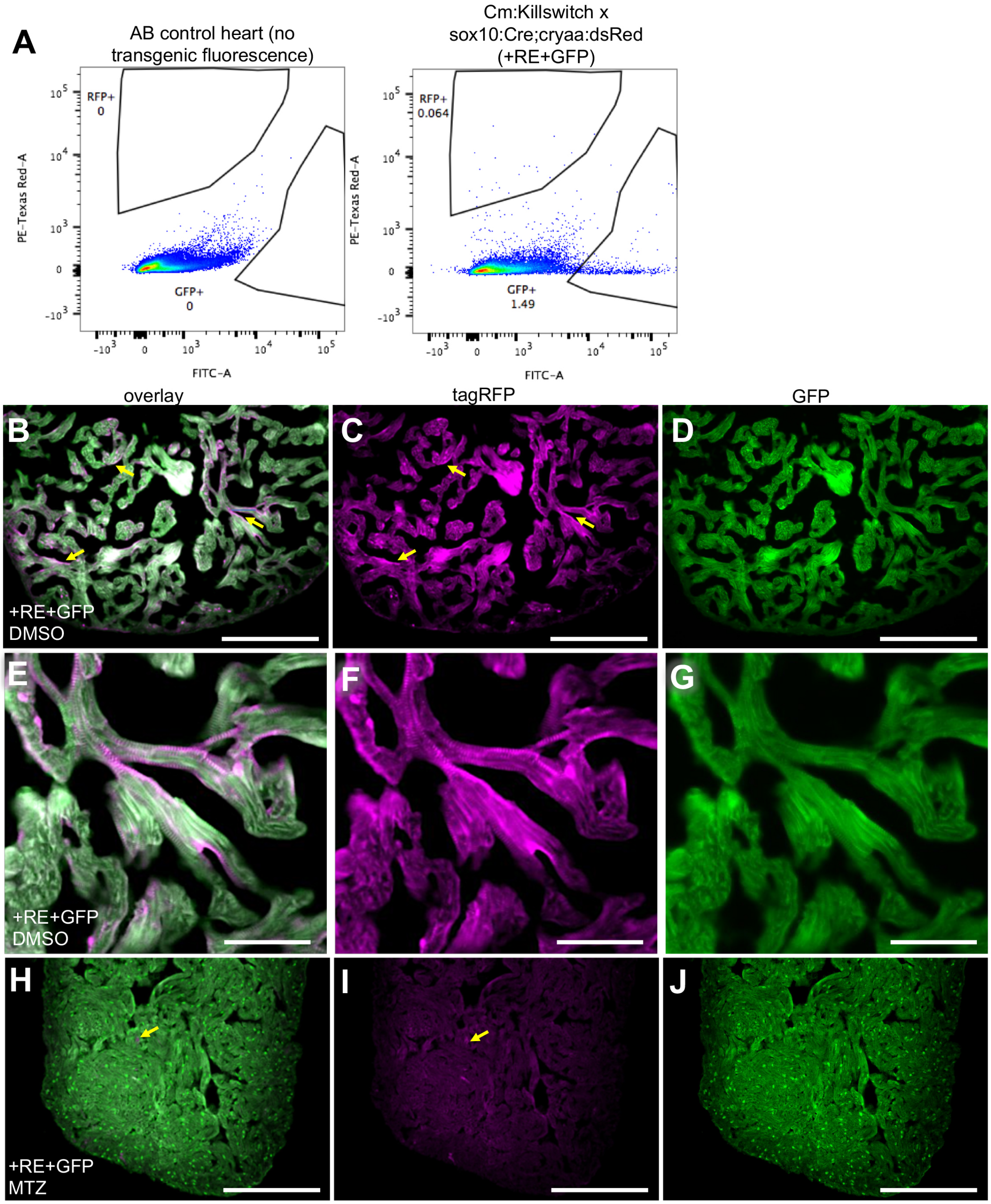

Supplementary Figure 10. NC-Cm labeling in adult transgenic fish. A) Flow cytometry analysis and quantification of labeled cardiomyocytes in *Cm:KillSwitch x Tg(sox10:cre;cryaa:dsRed)* individual

adult heart versus AB control. GFP and RFP gates were drawn based on non-transgenic/non-fluorescent AB wild-type hearts. Dissociated cells were gated on viability (DAPI) and singlets before analyzing GFP and RFP populations. Three or more individual hearts were similarly analyzed by flow cytometry to quantify numbers of GFP+ and RFP+ cardiomyocytes. GFP+ and RFP+ percentages were added to quantify total number of cardiomyocytes in a whole heart and then RFP+ numbers were divided by this to generate their percent contribution to the total adult cardiomyocyte population. The average values of these quantifications are listed in Figure 4A and C. Heart dissociation protocol was carried out as previously described[2]. B-D) Adult sections of ventricles from control (+RE+GFP, DMSO). Sections were stained with anti-tagRFP and anti-GFP antibody. Fluorescent images were captured at 20X magnification. Yellow arrows point to tagRFP+ NC-Cms in trabeculae of adult heart. E-G) Higher magnification of tagRFP+ trabeculae from control hearts as in B-D. Scale bar =50uM H-J) NC-Cm ablated (+RE+GFP, MTZ) heart sections and example of remnant tagRFP (yellow arrow) seen in the NC-Cm ablated ventricle sections. Scale Bar = 200uM.

Supplementary Figure 11.

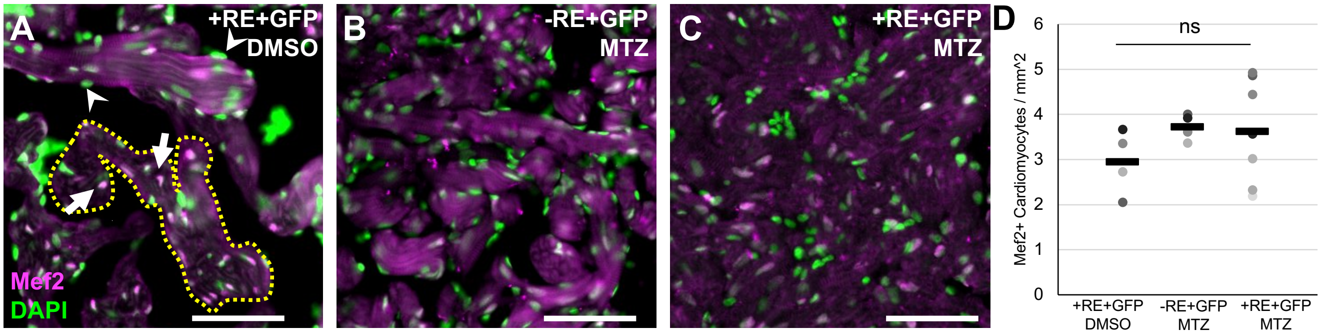

Supplementary Figure 11. Quantification of cardiomyocyte number and cell size in adult hearts. A-C) Examples of microscopy sections of ventricles stained with Mef2 and DAPI. Scale bar =15uM. Nuclei that are both Mef2 and DAPI positive (arrows in A) were used to count the number of cardiomyocytes per area defined by a ROI selection of the auto fluorescent trabecular myocardium, an example ROI selection is shown by yellow dashed line. Non-cardiomyocyte nuclei that were green only (arrowheads in A) were not counted. D) Cardiomyocyte numbers quantified from microscopy sections. Dots represent individual adult section measurements and bars the mean of each sample. ns = not significant from standard two-way T-test.

Supplementary Figure 12.

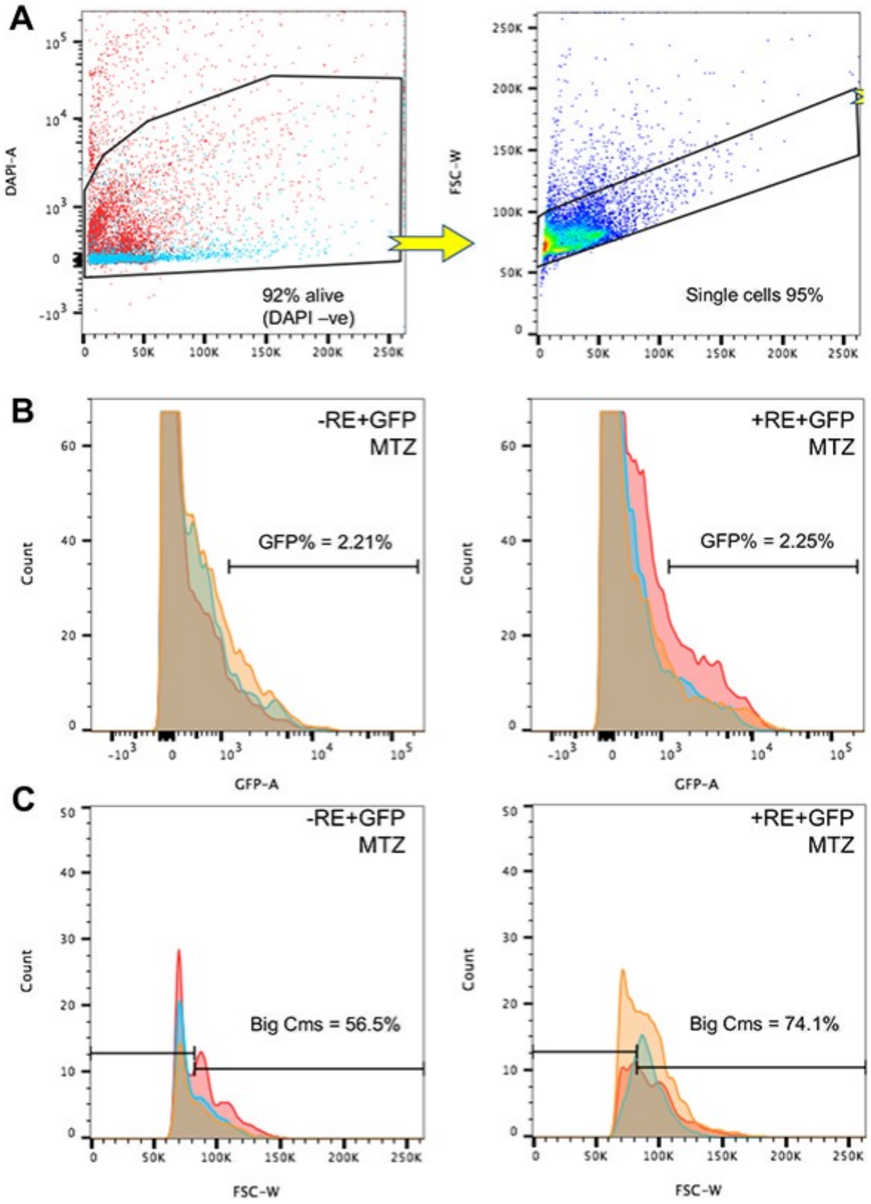

Supplementary Figure 12. Flow cytometry analysis of NC-Cm ablated and sibling control dissociated ventricles. A) Flow cytometry gating and analysis strategy for dissociate ventricle cells. Cells were first gated based on viability using DAPI staining, followed by selection for single cells as opposed to doublets based on the forward scatter width profile. Cells colored in blue represent the negative control for gating analysis, except for in the single cell gate. Percentages are averages of three or more

individual samples in the full experimental analysis. B) Cell populations from the single cell gate in panel A were analyzed for their percentage of GFP positive cells by histogram analysis and gating in the GFP channel, using the negative control sample to set the GFP+ gate. Percentages shown are averages from $n \geq 3$ individual, dissociated ventricle analysis in each sample and were not significantly different ($p=0.93$, standard t-test, control = left and NC-Cm ablated = right). Colors in the histogram represent individuals in each sample analysis and overlaid onto a single plot. C) GFP positive cells as determined from gating analysis in panel B were then analyzed for their size spectrum using the forward scatter width channel. The number of cells in the larger forward scatter width profile was not as prominent in the NC-Cm ablated samples (right panel). Thus, a gate was created based on the control forward scatter width profile to quantify the amount of larger GFP positive cardiomyocytes ('Big Cm's'gate). A significantly increased proportion of large GFP+ cardiomyocytes were found in the NC-Cm ablated (+RE+GFP, MTZ) ventricle samples compared to control ($p=0.009$, standard t-test, percentages shown are averages of $n \geq 3$ in each sample, colors represent individuals).

Supplementary Figure 13.

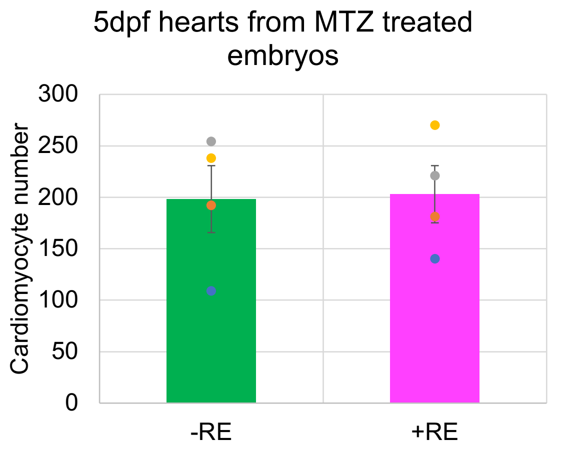

Supplementary Figure 13. Cardiomyocyte number is not significantly different between 5dpf NC-Cm ablated hearts (+RE) and sibling controls (-RE). Cardiomyocytes were counted by DAPI and GFP overlap in experimental embryos. Dots = individuals. Bar = Mean. Error bars = Standard Deviation.

Supplementary Table 1. Primers and Probes used in qPCR.

jag2b F	TGC TCG CAT CAC CCT TAT TT
jag2b R	AGG TCA CAC AGA ACC AAC AG
jag2b probe	CA CAG GAA CG ACG GTG GAG AAT GT
nrg2a F	GGC CAA TGG ACC CAA TCA
nrg2a R	TGC TCC GTG CCG AAT TAC
nrg2a probe	TG GTC CTG AGG AGA TTC CCA TGG TA
myl7 F	GCA CAG ACC CAG AGG AAA C
myl7 R	GGT CAT TAG CAG CCT CTT GAA
myl7 probe	CG ACC CTA AT GCC ACA GGA GTT GT
Rpl11 F	CATTGGAATCTACGGATTGGA
Rpl11 R	TGATGCCGTCATACTTCTGC
Rpl11 probe	CCGGTTCAGCATTGCTGACAAA

Supplementary References:
1. Tessadori, F. *et al.* Identification and Functional Characterization of Cardiac Pacemaker Cells in Zebrafish. *PLoS One* **7,** e47644 (2012).
2. Sander, V., Suñe, G., Jopling, C., Morera, C. & Belmonte, J. C. I. Isolation and in vitro culture of primary cardiomyocytes from adult zebrafish hearts. *Nat. Protoc.* **8,** 800–809 (2013).
