# Supplementary material for: Loss of embryonic neural crest derived cardiomyocytes causes adult onset hypertrophic cardiomyopathy in zebrafish
Source: Nat Commun. 2018 Nov 2;9:4603. doi: 10.1038/s41467-018-07054-8 (PMC6214924; doi:10.1038/s41467-018-07054-8)
Supplement: Supplementary file 8 — Description of Additional Supplementary Files [file 41467_2018_7054_MOESM8_ESM.docx]

**Title:** Supplementary Movie 1.
**Description:** 3D reconstruction of a 3dpf heart from a transgenic embryo of sox10:cre;cryaa:dsRed and Cm:KillSwitch. NC-Cms are labeled in magenta and all other CMs are labeled in green.

**Title:** Supplementary Movie 2.
**Description:** 3D reconstruction of the ventricle of a 3dpf, Tp1:GFP line crossed to sox10:tagRFP line. Sox10 positive cells are labeled in magenta and Tp1 positive cells i.e. Notch activity positive cells are labelled in green.

**Title:** Supplementary Movie 3.
**Description:** 3D reconstruction movie of a control adult cardiomyocyte seen in Fig.4L.

**Title:** Supplementary Movie 4.
**Description:** 3D reconstruction of NC-Cm ablated adult cardiomyocytes seen in Fig.4M.

**Title:** Supplementary Movie 5.
**Description:** Example of an adult fish undergoing the swim trial test and collapsing to fatigue
